# Quality of dying and death desired by residents of Kagawa Prefecture, Japan: a qualitative study

**DOI:** 10.1186/s12199-019-0806-8

**Published:** 2019-07-31

**Authors:** Kanae Kanda, Nobuko Takashima, Yoshimi Tsuji, Katsunori Yokoyama, Tomohiro Hirao

**Affiliations:** 1 Department of Public Health, Faculty of Medicine, Kagawa University, 1750-1 Ikenobe Miki-cho Kita-gun, Kagawa, 761-0793 Japan; 2 Department of Nursing, Faculty of Health Sciences, Kagawa Prefectural University of Health Sciences, Kagawa, Japan; 3 Division of Health and Welfare Administration, Department of Health and Welfare, Kagawa Prefectural Office, Kagawa, Japan

**Keywords:** Group interview, Local residents, Quality of death, Quality of dying

## Abstract

**Background:**

Achieving a desirable death is an urgent aging-related problem in Japan. However, measures of the quality of death and dying in Japan are lacking. This study aimed to identify components of a desirable death in the residents of Kagawa prefecture, Japan, through focus group interviews.

**Methods:**

A group interview was conducted with 30 residents aged 20–80 (*M*_age_ = 50.9, *SD* = 22.1 years; 43.3% ≥ 65 years; 40.0% unemployed) who had experienced the death of a closely associated person. Participants were grouped into four generations with diverse characteristics (e.g., age, sex, occupation). The interview lasted 1–2 h and involved one interviewer, one observer, and one recorder. The interview theme was “What is a desirable death?” Participants were asked “What do you want to achieve before you die?” or “What would a close friend want to experience when death is near?” We then extracted important items related to “desirable death” using serialization and observation records, while also consulting three analysts. The analysis results of the four generations were ultimately integrated into final categories.

**Results:**

The most common experience of a familiar death was that of parents, followed by grandparents. Half of participants had witnessed the death. Through category analysis, eight important categories related to desirable death were ultimately extracted. Nine items were identified as common to all generations. While the elderly generation had wide-ranging opinions, the younger generations’ opinions tended to concentrate on satisfaction with life and family relations.

**Conclusion:**

Eight concepts were extracted as important factors of a desirable death from the residents of Kagawa prefecture, Japan.

## Background

The quality of dying and/or death (QOD) refers to a dying person’s views on what constitutes a “desirable death.” It is important to understand people’s views on what constitute the components of a desirable death. Patrick et al. [1] reported six components of terminal QOD: (1) symptoms and personal care, (2) preparation for end of life, (3) moment of death, (4) family, (5) treatment preferences, and (6) whole-person concerns, meaning, and purpose. It seems that “desirable death” is influenced by cultural background and differences in medical systems. In Japan’s population ages, there is growing interest in how elderly adults die. However, the traditional culture keeps death separate from everyday life. In addition, it is ethically problematic to investigate patients at the terminal stage or to conduct questionnaire surveys with newly bereaved families. In Japan, the number of deaths of elderly adults will increase rapidly in the future, raising a major social need to consider what constitutes a “desirable death” and the quality of death at an early stage.

Research on QOD began in the USA around 1980 [2–5] and has increased since 2000 [6, 7]. Indeed, searching PubMed using “quality of dying” as a keyword retrieved about 32,000 hits as of the writing of this article. Many of these are research studies conducted in western countries. In addition, *The Economist*, a UK-based magazine, announced worldwide rankings in the 2015 Quality of Death Index [8]. Regarding measurement of QOD, a systematic review of measures by Hales et al. [9] found that of the six published measures reviewed, the Quality of Dying and Death questionnaire (QODD) [10, 11] was the most widely studied and best validated. However, the QODD contains questions that are unsuitable for Japanese people, such as questions about religion, and there are problems with ease of use. Miyashita and colleagues’ Good Death Inventory (GDI) is the only measure that has been developed in Japan [12, 13]. The GDI is aimed at bereaved families of end-stage cancer patients and is specialized for use in terminal care at medical facilities. Therefore, it has limitations in that it does not include non-medical items (e.g., economic conditions, worries about living expenses, wills, inheritance issues, funerals).

Focusing on this topic, we recognized the need to develop a scale that could be used to consider QOD when death is approaching. Although it is important for bereaved families to evaluate patients’ QOD, it is also necessary to prepare in advance of them reaching the end of life by knowing the conditions for people to achieve a desirable death.

Therefore, as a pilot study, we undertook to extract important elements of desirable death from interview surveys divided by age groups in small-scale community panels.

## Methods

### Design

We used a focus group interview to identify the attributes of a desirable death. This is a common data collection technique for exploratory qualitative studies to generate hypotheses and provide rich descriptive information about a phenomenon [14, 15]. Researchers do not impose theoretical assumptions a priori but instead let participants frame questions from the “ground up.”

### Participants

The study included men and women aged 20–80 years dwelling in communities in Kagawa from March to February 2017. A group interview was conducted for a total of 30 people divided into four generations: (1) 20–29 years old, (2) 30–39 years old, (3) 40–59 years old, and (4) 65–80 years old. We did not use random sampling as our study focused primarily on obtaining meaningful and broad qualitative information from many cases, rather than sourcing quantitative data from a sample representative of the general population. To obtain deeper and wider information on the subject of “what is a desirable death,” we asked the welfare commissioner and public health nurses in the municipality, who are familiar with the area, to assist with the recruitment, and we gathered local residents of five areas. We recruited participants who were local residents; had diverse backgrounds in terms of age, gender, occupation, residential area, and care environment; were familiar with cases related to the theme; and were able to clearly discuss these cases. Before being interviewed, participants were informed of the contents of the study and provided written consent.

### Focus group interview

The focus group interview guide is shown in Table 1. The interview location was a quiet private room containing one interviewer, one observer, and a recorder [16]. The interview lasted 1 to 2 h. The interview theme was “What is a desirable death?” According to the interview guide, this theme was explored using two types of questions. The first concerned their experience of a familiar person’s death, and the second concerned their thoughts about a “desirable death.” Specific questions included “What do you want to achieve before you die?”; “What would a close family or friend want to happen when death is near?”; and “What conditions make a desirable death?”

**Table 1:** Focus group interview guide

(1) About one’s experience of a familiar person’s death	
Have you experienced loss of a family member or another familiar person in the past?	
Did you take care of those who died?	
Please tell us in detail about your impressions of the end-of-life care in these cases.	
Have you had had trouble or difficulty in end-of-life care?	
On the contrary, was there something good or happy that happened?	
(2) Your thoughts about “desirable death”	
What do you want to achieve by the time you die?	
What does a close family member or friend want to happen when death is near?	
What conditions would make a desirable death?	
What are your thoughts with respect to satisfaction and self-determination of medical care?	
What do you think about the cause of death and whether the family feels it was a convincing death?	

We asked focus group participants to discuss their experiences with the deaths of family members, friends, or patients and to reflect on what made those deaths good or bad. When necessary, we asked probing questions to clarify a comment or obtain more detail [17].

### Data analysis

The following techniques were used to analyze the focus group interviews. Multiple analysts performed the same task, discussed similarities and differences, collaboratively developed the most objective explanations, and reached consensus on the important categories. In the analysis, important items related to a “desirable death” were extracted using serialization of words and observation records while consulting with three analysts, and the important categories were organized. The items and categories extracted from the five groups were ultimately integrated to identify the final categories [18, 19].

The concrete steps of the primary analysis were as follows: prepare a verbatim record of the interview, extract the content related to a “desirable death” interview item, and then summarize and code that content. In addition to the verbatim transcripts generated from the electronic audio records, we considered participants’ response to the sequential observation record, and important items (words) reflecting QOD were extracted while being confirmed by the analysts. The secondary analysis focused on identifying similarities in important items (i.e., words) obtained through the primary analysis and subcategorizing them. These subcategories were further categorized, and important categories were extracted. In the tertiary analysis, important items and important categories of each group were integrated, and common points and differences according to generation were examined (combined analysis). Through these processes, the final important categories were extracted. The analysis was performed by three analysts, who discussed all common points and differences, and decided on important categories through consensus.

### Ethics statement

Informed consent was obtained from the participants. In addition, this study was approved by the Ethics Committee of Kagawa University Faculty of Medicine and Graduate School of Medicine (approval number: Heisei28-113).

## Results

The characteristics of the study participants are shown in Table 2. Focus group interview participants ranged in age from 20 to 80 years (*M*_age_ = 51 years, *SD* = 22.1 years), 73% were women, 43% were 65 years old or older, and 40% were unemployed. The most frequently mentioned experience of death of a familiar person referred to parents, followed by grandparents. Half of the participants had witnessed the person dying.

**Table 2:** Baseline characteristics of the study participants

Characteristics	*n*	%
Sex		
Male	8	26.7
Female	22	73.3
Age (years)		
20–29	6	20.0
30–39	6	20.0
40–59	5	16.7
65–80	13	43.3
Employment		
Unemployed	12	40.0
Student	6	20.0
Full time	8	26.7
Part time	3	10.0
Other	1	3.3
Family structure		
Single person	4	13.3
1 generation (couple)	9	30.0
2 generations (parent and child)	11	33.3
3 generations (parent and child, grandparents)	6	20.0
Other	0	0.0
Experience of familiar person’s death		
Yes	30	100.0
No	0	0.0
Time interval between familiar person's death and survey (years)		
< 1	4	13.3
1–2	9	30.0
3–4	6	20.0
≧ 5	11	36.7
Relationship to familiar person who died (multiple answers)		
Parents	11	34.4
Grandparents	10	31.3
Husband/wife	4	12.5
Child	0	0.0
Brother/sister	2	6.3
Friend	4	12.5
Other	1	3.1
Experience of attending the deathbed		
Yes	15	50.0
No	15	50.0

Regarding category analysis, 53 important items were extracted via the primary analysis. The secondary analysis produced 19 subcategories. In the tertiary analysis, eight important final categories were extracted (Table 3). The eight final categories were named as follows: (1) preparation for death, (2) satisfaction with life, (3) reliable medical environment, (4) good family relationship, (5) independence for oneself, (6) no physical and psychological distress, (7) dying in a favorite place, and (8) attending the deathbed.

**Table 3:** Eight important categories of “Desirable Death” and nine important items common to all generations

*8 final categories* *(tertiary analysis)*	*19 sub-categories* *(secondary analysis)*	*53 important items* *(primary analysis)*	Age (years)			
			65–80	40–59	30–39	20–29
(1) Preparation for death	Know medical condition and life expectancy	Know the disease/condition and life expectancy	●	●		
	Be prepared for and accept death	Both patient and family are accepting of death (*)	●	●	●	●
		Family can accept death (after it occurs)	●	●	●	
		To be dead according to the thought of surrounding people (surrounding people's agree)		●	●	
	Leave thoughts in a will and testament	Do not have to worry about death	●			
		Thinking about own death in daily life		●		●
		Write the ending notes, wills, or testaments	●		●	
(2) Satisfaction with life	To feel that one has lived until fulfilling one’s life purpose (i.e., not dying prematurely)	Not die earlier than expected (time of death must be as predicted)	●			
		To feel that one has lived until fulfilling one’s life purpose (i.e., not dying prematurely) (*)	●	●	●	●
	Having no regrets	Having no regrets (*)	●	●	●	●
		I think I did something I wanted to do				●
	Mourning one’s/your death	Mourning one’s/your death	●		●	
		A life that can be remembered even after death			●	●
(3) Reliable medical environment	Receiving enough treatment	Discussing end-of-life medical care to be provided	●			
		Receiving enough treatment	●	●		
		Participating in decisions about treatment	●			
		Not being treated to prolong life	●		●	
		Being able to choose dignity in death			●	
	Reliable medical environment and staff	A safe medical environment is in place	●			
		Having a reliable doctor nearby	●			
(4) Good family relationship	Having family support and people around	Having someone to count on	●			●
		A person with whom you can be vulnerable will care for you until the end			●	●
	Both patient and family consent to enough nursing care	Not being a burden to family members (*)	●	●	●	●
		Care period is not long	●		●	
		Good relationship with family of nursing care (*)	●	●	●	●
		Family members can proactively provide nursing care		●		
		Receiving enough nursing care	●	●	●	
(5) Independence for oneself	Being able to do what one hoped at the end	Being able to do what one hoped at the end	●		●	
		Living as usual until the end	●			
		Having fun living	●			
	Independence for oneself in daily activities	Eat by myself until the end	●			
		Go to the bathroom by myself	●	●		
	Having intention to communicate and move until just before death	Having intention until death (consciousness/communication)	●			
		Being fine until just before death	●			
		Bedridden time is short	●	●		
		No dementia	●	●		
(6) No physical and psychological distress	Being free from physical distress	Being free from physical distress (*)	●	●	●	●
		Not suffering breathing difficulties	●			
	Being free from emotional distress	Not angry or complaining			●	
		Being free from emotional distress (*)	●	●	●	●
(7) Dying in a favorite place	Being able to stay in one’s favorite place	Being able to stay in one’s favorite place (*)	●	●	●	●
	Feel at ease in the environment	Spending time in places and with people without hesitation	●			
		Spend end of life with the family	●			
		Good environment around the deathbed		●		
(8) Attending the deathbed	Seeing people whom one wants to see	Seeing people whom one wants to see	●		●	
	Saying important things to dear people	Expressing thanks to people	●			
		The family can tell the patient what they want to say	●	●		
		Telling dear people what one wants to say		●		●
	Family present at the deathbed	Family rushes to the deathbed (including those who live far away)	●		●	
		To die surrounded by family		●		
		Family present at the deathbed (*)	●	●	●	●
	To die unexpectedly and effortlessly	To die unexpectedly and effortlessly	●		●	
		Not sudden death	●		●	

*Items for measuring the desired death*

How important do you consider each of the following items for a desirable death? Please place the appropriate number next to each statement: 1 = absolutely disagree, 2 = disagree, 3 = neither agree nor disagree, 4 = agree, and 5 = absolutely agree

*Nine important items common to all generations (extracted via primary analysis)

●Important items were extracted

In the tertiary analysis, regarding the characteristics of the age groups, while the opinions of the elderly generation were abundant and multifaceted, the opinions of the younger generation tended to concentrate on satisfaction with life and family relations. For participants in their twenties and thirties, opinions on trust in medical care and independence or consciousness were not expressed.

Nine of the extracted items were common to all generations: “both patients and family are accepting of death,” “To feel that one has lived until fulfilling one’s life purpose (i.e., not dying prematurely),” “having no regrets,” “not being a burden to family members,” “good relationship with family of nursing care,” “being free from physical distress,” “being free from emotional distress,” “being able to stay at one’s favorite place,” and “family present at the deathbed” (Table 3).

## Discussion

In this research, we conducted an interview survey with Japanese residents and extracted factors related to a desirable death. The first major contribution of this study is that it explored the raw opinions of local residents using a focus group interview. This addresses gaps in past research, which primarily used the QODD (which contains questions that are not suitable for Japanese people) and GDI (which does not include non-medical items or consider terminal care outside of medical facilities). We found that the contents of the important categories were almost the same as those of previous studies in Japan, but differences were found in the following points. Compared to Miyashita et al.’s [13] GDI, new items identified in our survey were “prepare for death” and “to have a death surrounded by family members.” Other new items included “the patients and family are prepared for death,” “missed the moment of death,” “being able to communicate and move until just before dying,” and “to suddenly die in a healthy state.” In addition, compared with the QODD scale of Curtis et al. [10], our survey found 13 items in common. These common items included “control of pain and respiration,” “can eat and excrete by yourself,” “to do what you want to do,” “spend time with your family and have a chance to say goodbye,” “discussion about treatment,” “peaceful death,” “feeling of no burden on family members,” “not doing life extension treatment,” “desired place,” and “surrounded by family.”

The second major contribution of this study was that by conducting a wide range of interview surveys by age, we were able to clarify the generational factors. Nine items in this study spanned all generations. Miyashita et al.’s [12] survey of Japanese people over 40 years old identified 10 core domains as factors of a “desirable death” that Japanese people commonly think are important. Our study covered participants aged 20–80 years, and when comparing the nine items that were common among all generations with the items found by Miyashita et al., eight were consistent. These items focused on “physical and psychological comfort,” “good relationship with family,” and “feeling that one’s life was complete.” The remaining item in our study was entirely new (“the patients and family are prepared for death”).

Regarding characteristics related to age, the opinions of the elderly generation were multifaceted and referred to specific diseases, treatment, physical condition, psychological state, family relationship, preparation for death, and physical environment. Participants in their twenties and thirties, on the other hand, focused on psychological conditions such as “satisfaction with life” and “acceptance of death” and family relations such as “how to spend time with family” and “sense of burden of nursing care.”

This study has some limitations. First, since a focus group interview was used, the sample size was naturally small. Second, because the study area was limited, we cannot make inferences about regional differences in our results. Given both of these factors, the sample cannot be considered representative of the general Japanese population [20]. Therefore, we need to build on this study’s results through a large-scale survey. In future research, we intend to use the items extracted in this study to conduct a questionnaire survey on a scale that can reflect the Japanese population. The results could then be analyzed to determine the weight of each survey item and refine the rating scale that to comprise no more than 10 items. The purpose of this series of studies is to help all Japanese achieve their desirable death. As existing assessment measures are mainly applicable to bereaved families, we want to develop an objective indicator of QOD that assesses the manner of death desired by living individuals and their families, and their ideal form of terminal care, rather than assessing bereaved families only.

## Conclusions

Eight concepts were extracted as important factors of a desirable death identified by members of the general Japanese population: (1) preparation for death, (2) satisfaction with life, (3) reliable medical environment, (4) good family relationship, (5) independence for oneself, (6) physical and psychological distress, (7) dying in a favorite place, and (8) attending the deathbed.

## Data Availability

The datasets supporting the conclusions of this article have been included within the article and its additional files.

## Abbreviations

QOD: Quality of death
QODD: Quality of Dying and Death questionnaire
GDI: Good Death Inventory
